# Comparison of Urinary Sodium and Blood Pressure Relationship From the Spot Versus 24‐Hour Urine Samples

**DOI:** 10.1161/JAHA.119.013287

**Published:** 2019-10-16

**Authors:** Abu Mohd Naser, Mahbubur Rahman, Leanne Unicomb, Solaiman Doza, Shuchi Anand, Howard H. Chang, Stephen P. Luby, Thomas F. Clasen, K.M. Venkat Narayan

**Affiliations:** ^1^ Department of Environmental Health Sciences Rollins School of Public Health Emory University Atlanta GA; ^2^ Emory Global Diabetes Research Center Hubert Department of Global Health Rollins School of Public Health Emory University Atlanta GA; ^3^ International Centre for Diarrhoeal Disease Research Bangladesh (icddr,b) Dhaka Bangladesh; ^4^ Division of Nephrology School of Medicine Stanford University Stanford CA; ^5^ Department of Biostatistics and Bioinformatics Rollins School of Public Health Emory University Atlanta GA; ^6^ Woods Institute for the Environment Stanford University Stanford CA

**Keywords:** 24‐hour urine, blood pressure, salt intake, sodium, spot urine, urinary sodium, High Blood Pressure, Hypertension, Epidemiology

## Abstract

**Background:**

We compared the relationship between sodium (Na) intake and blood pressure when Na intake was estimated from first‐ and second‐morning spot urine samples using the INTERSALT (International Study on Salt and Blood Pressure) formula, versus directly measured 24‐hour samples.

**Methods and Results:**

We collected 24‐hour urine and first‐ and second‐morning voids of 383 participants in coastal Bangladesh for 2 visits. We measured participants’ blood pressure using an Omron^®^ HEM–907 monitor. To assess the shape of the relationship between urinary Na and blood pressure, we created restricted cubic spline plots adjusted for age, sex, body mass index, smoking and alcohol consumption, physical activities, religion, sleep hours, and household wealth. To assess multicollinearity, we reported variance inflation factors, tolerances, and Leamer's and Klein's statistics following linear regression models. The mean daily urinary Na was 122 (SD 26) mmol/d for the first; 122 (SD 27) mmol/d for the second; and 134 (SD 70) mmol/d for the 24‐hour samples. The restricted cubic spline plots illustrated no association between first‐morning urinary Na and systolic blood pressure until the 90th percentile distribution followed by a downward relationship; a nonlinear inverse‐V‐shaped relationship between second‐morning urinary Na and systolic blood pressure; and a monotonic upward relationship between 24‐hour urinary Na and systolic blood pressure. We found no evidence of multicollinearity for the 24‐hour urinary Na model.

**Conclusions:**

The urinary Na and systolic blood pressure relationship varied for 3 urinary Na measurements. Twenty‐four‐hour urinary Na captured more variability of Na intake compared with spot urine samples, and its regression models were not affected by multicollinearity.

**Clinical Perspective**

**What Is New?**

- Twenty‐four‐hour urinary samples capture more variability of daily sodium intake compared with daily sodium intake estimated from spot urine samples.
- Different shapes of the relationship between sodium intake and blood pressure could be identified from the same population if sodium measurements come from 24‐hour versus spot urine samples.

**What Are the Clinical Implications?**

- As per the sodium intake and blood pressure relationship from 24‐hour urine samples, reduction in sodium intake may reduce blood pressure of hypertensive patients.

## Introduction

High systolic blood pressure (SBP) is the largest contributor to the global disease burden that accounts for annual 10.4 million deaths and 218 million disability‐adjusted life years globally.^1^ High intake of dietary sodium (Na) is an important modifiable risk factor for high BP^2^ and other related cardiovascular diseases such as stroke and myocardial infarction.^3^, ^4^ High Na intake accounts for a greater proportion of the global disease burden than tuberculosis,^5^, ^6^ and long‐term, population‐level reductions in Na intake is a priority for achieving global health targets (eg, 25% reduction in premature mortality from noncommunicable diseases by 2025).^7^ Yet, considerable controversies exist regarding the appropriate strategies to measure Na intake in epidemiological studies, partly because some studies suggest low Na intake may increase cardiovascular disease risks.^8^, ^9^

An average 93% of ingested daily Na is excreted in 24‐hour urine samples.^10^ One strategy to measure the daily dietary Na intake is to measure urinary Na concentrations. Both spot urine and 24‐hour urine samples are used to determine the daily dietary Na intake.^11^ Spot urine samples are logistically convenient; however, estimation from spot urine samples are affected by Na content of recently ingested food and diurnal excretion patterns.^11^ Twenty‐four‐hour urine collection is the recommended method of measuring Na intake in epidemiological studies but is logistically difficult and burdensome for participants at the population level.^12^, ^13^, ^14^ Nevertheless, urinary Na excretion exhibits a weekly rhythm even at constant daily Na intake, which is regulated by aldosterone and cortisol hormone.^15^ Therefore, a single measurement of 24‐hour urinary Na is not sufficient for capturing the long‐term variation of Na intake in a population, and hence, multiple 24‐hour urine sample collection is recommended for determining long‐term Na intake.^16^, ^17^

Pooled analyses from the multicountry PURE (Prospective Urban Rural Epidemiology) study that relied upon estimated daily urinary Na from spot urine suggest a J‐shaped relation between urinary Na and cardiovascular mortality.^18^ These analyses suggest low daily Na intake below 193 mmol/d (or 4.43 g/d) may increase the cardiovascular risks of the population, and contradict the World Health Organization's recommendation to lower Na intake below 87 mmol/d.^19^ Nevertheless, studies that directly measured 24‐hour urinary Na found a linear positive association between Na intake and cardiovascular mortality.^20^, ^21^, ^22^ The controversies of Na intake and cardiovascular diseases relationship can be better studied if both spot and 24‐hour urine samples of an individual are compared to establish such a relationship. The objective of our analyses is to compare and explain the nature of the relationship between Na intake and BP when 3 urinary samples for estimating daily urinary Na excretion are used—spot first and second morning urine Na samples versus 24‐hour urine samples.

## Methods

### Data Sources and Study Setting

**Figure 1:**
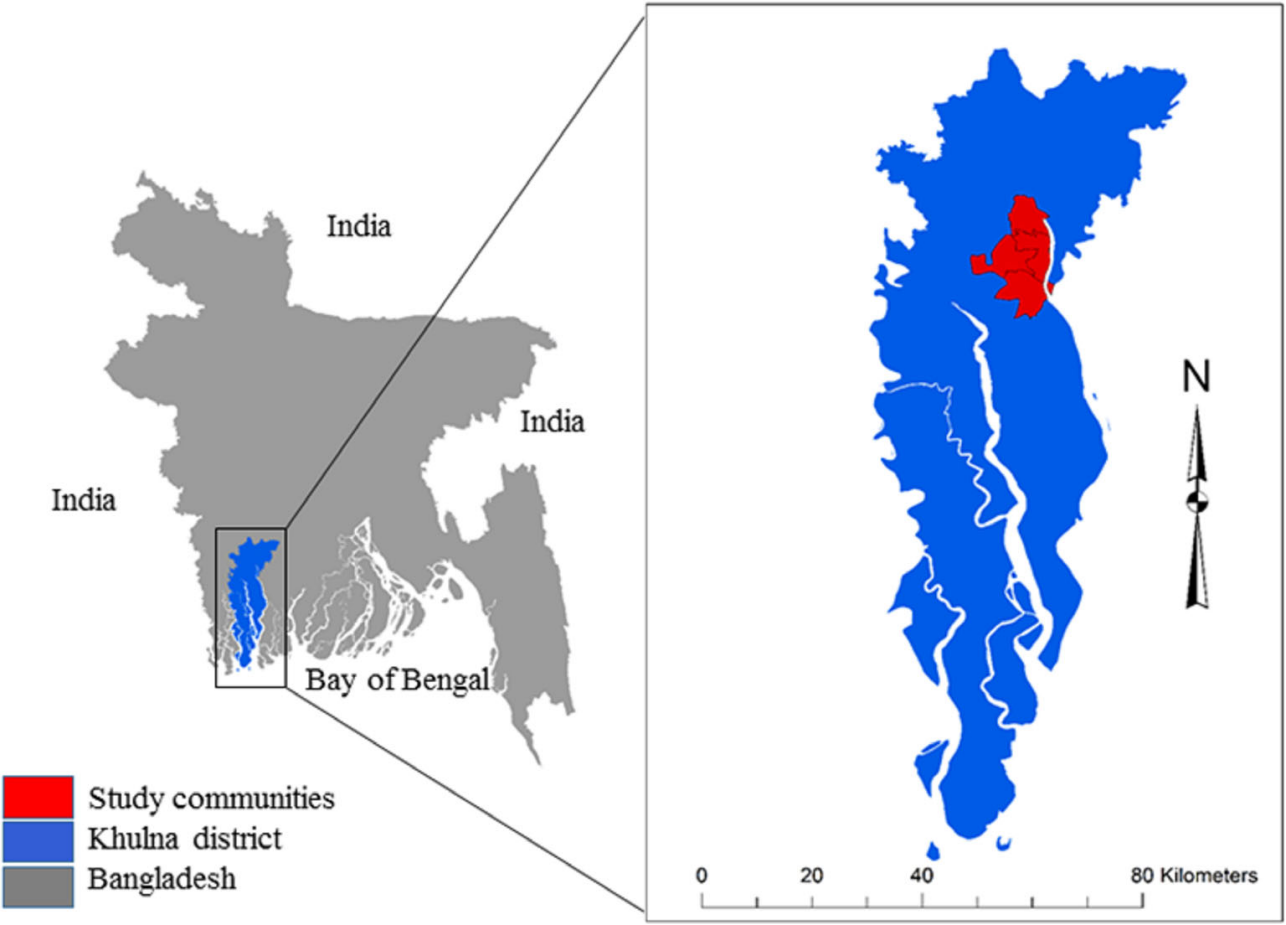
Study location in Khulna district of southwest coastal Bangladesh.

The data that support the findings of this study are available from the corresponding author upon reasonable request. Data for this article came from southwest coastal Bangladesh, where the population has high Na intake through their drinking water sources.^23^, ^24^ Seawater intrusion–induced water salinity has increased the Na content of drinking water in this population. We used data from a cohort study conducted in 4 communities of Dacope and Batiaghata subdistricts of Khulna district (Figure 1). The study was conducted in preparation for a community‐based randomized controlled trial for the health impact evaluation of an intervention to reduce groundwater salinity.^25^ We conducted 2 visits among 383 participants who were ≥20 years of age from 166 households in each community during the premonsoon (May 10–June 21, 2016) and the monsoon (July 21–August 21, 2016) periods. The objective of the study was to assess whether spot urine Na from participants’ first or second morning urine samples can be relied upon as a good proxy of the daily Na intake as opposed to Na measured from the 24‐hour urinary samples.

### Cardiovascular Risk Factor Data

We collected data using a structured questionnaire on household assets, demographics, height, weight, participant‐reported smoking status (never smoker, current smoker, and former smoker), and alcohol consumption during the first visit. We also collected participants’ self‐reported information about work‐related physical activity (vigorous physical activities, moderate physical activity, and sedentary activity), hours of sleep, religion, households’ use of table salt for cooking, and participants’ additional consumption of table salt during the meal. We used the World Health Organization Global Physical Activity Questionnaire for determining the status of physical activities among the participants.^26^ Participants’ weight in kg was measured at both visits using a Seca weight machine (Model: 874‐1321009; accuracy: 0.05–0.1 kg, Hamburg, Germany) and height at the first visit using a Shorr board (accuracy: 1/8 inch or 0.1 cm; Olney, Maryland). We derived the household wealth score by principal component analysis using the household asset data for ownership of refrigerator, television, mobile phones, motorcycle, bicycle, sewing machine, chair, table, wristwatch, wardrobe, wooden cot, motor pump, rice husking machine, motorized rickshaw, car, and access to electricity.^27^

### Urine Sample Collection and Analysis

Each participant received a 4‐L plastic container for 24‐hour urine collection, two 15‐mL tubes for first and second morning urine collections, and a plastic mug to collect the voided urine and transfer to the container and tubes. On day 1, following standardized 24‐hour urine collection protocols, participants started by collecting the second morning void. Participants were instructed to transfer a portion of second morning void to a 15‐mL tube, and the remaining to the 4‐L plastic container. They were instructed to transfer all other voids of the day and night to the 4‐L plastic container, and transfer the portion of next morning's first void in another 15‐mL tube and remaining to the 4‐L plastic container. Therefore, first morning void was collected on the second day and vice versa. The volume of the 24‐hour collected urine was measured at household‐level by research assistants, and a 15‐mL sample from the 4‐L plastic container was taken after stirring. All urine samples were transported to a field laboratory at 2 to 8°C for processing and analysis on the same day. The Direct Ion Selective Electrode method^28^ was used for urinary Na and potassium (K) measurements using a semi‐auto electrolyte analyzer (Biolyte2000, Bio‐care Corporation, Taiwan, coefficient of variation: ±5%). We measured urine creatinine by a colorimetric method (Jaffe reaction).

We multiplied 24‐hour urine volume with the urinary concentrations of Na and creatinine from the 24‐hour sample to get the directly measured 24‐hour urinary Na and creatinine excretion. We used the INTERSALT (International Study on Salt and Blood Pressure) formula to estimate daily urinary Na from the first and second morning urine samples.^29^ INTERSALT formula was used by previous epidemiological studies in salinity‐affected southwest coastal Bangladesh to estimate the daily Na intake.^24^, ^30^ The INTERSALT formula was developed from a population‐based study conducted in 52 population groups from 32 countries to evaluate the success of a population sodium reduction strategy.^31^ For men, the equation is ((25.46+0.46×Na_SPOT_)−2.75×Cr_SPOT_−0.13×K_SPOT_+4.10×bmi+0.26×age) where Na_SPOT_, Cr_SPOT_, and K_SPOT_ refer to sodium, creatinine, and potassium concentrations in spot urine samples. For women the equation is ((5.07+0.34×Na_SPOT_) −2.16×Cr_SPOT_−0.09×K_SPOT_+2.39×bmi+2.35×age−0.03×age^2^).^31^

### BP Measurement

On the same day of starting 24‐hour urine collection, participants’ BP was measured at their home using Omron^®^ HEM–907 (accuracy: within ±4 mm Hg, Kyoto, Japan) digital BP monitors between 7:30 am and 2:00 pm.^32^ BP was measured following World Health Organization guidelines for BP measurement^33^ and the recommendations described by Pickering et al 2005.^34^ Caffeine (tea, coffee, carbonated beverages), eating, heavy physical activities, and smoking were prohibited for 30 minutes before measuring BP. Participants rested for 5 minutes on a chair with both arms supported. An appropriate‐sized cuff was used based on mid‐upper arm circumference of the participants (small‐size cuff if mid‐upper arm circumference <22 cm; medium‐size cuff if mid‐upper arm circumference ≥22 to <32 cm; and large‐size cuff if cuff ≥32 cm). BP was measured 3 times: first left arm, then right arm, then again left arm. We used the arithmetic mean of 3 BP measurements in analyses.

### Statistical Analyses

We reported the descriptive statistics of the outcome and exposure variables and other covariates. We presented the histogram of 3 types of urinary Na measurement, and their unadjusted relationship with BP through scatter plots and median splines. We calculated the pairwise Pearson's correlation coefficients between each of the 3 urinary Na measurements and age, dichotomous sex, and body mass index (BMI) of the participants from pooled person‐visits of 2 visits (N=651). We calculated participant intraclass correlations for each of the 3 urinary Na measurements by 1‐way random‐effects models.^35^

Twenty‐four‐hour urine sample collections in population‐based studies may be affected by over‐ or undercollection, which may result in biased estimates. One indirect way of measuring whether the 24‐hour urine sample collections were complete is to rely on the creatinine index, which is defined as the ratio between measured and predicted urinary creatinine.^12^ Creatinine index ≥0.7 is suggestive of complete 24‐hour urine collection.^12^, ^36^ As mentioned earlier, we measured urinary creatinine concentrations by Jaffe reaction and also calculated predicted urinary creatinine by the Kawasaki formula.^37^ We calculated the creatinine index for all person‐visits and then additionally reported findings from the restricted analyses among the person‐visits that had complete 24‐hour urine collection measured by creatinine index ≥0.7.

To compare the shape and magnitude of the relationship between daily urinary Na measurements and BP, we considered 3 approaches of statistical modeling. First, to visually assess the shape of the relationship and to detect any nonlinear relationship, we plotted restricted cubic spline plots^38^ to illustrate the trajectories of BP with the increasing level of urinary Na concentrations. We used default 5 knots at equidistance percentiles (5th, 27.5th, 50th, 72.5th, and 95th) according to Harrell's rule to create the flexible smooth plots.^39^ Restricted cubic plots assume cubic polynomials in segments after the first knot and before the last knot.^40^ Hence, our spline plots could identify the nonlinear association between the fifth and 95th percentile distribution of urinary Na measurements. We then used the Wald test for detecting departure from linearity (*P*≤0.05 suggestive of nonlinear association).^41^

Secondly, we modeled urinary Na as categorical variables. We used tertiles of daily urinary Na measurements to create urinary Na categories. We then used multilevel linear models to determine the associations between tertiles 2 and 3 with BP compared with tertile 1. Thirdly, we modeled urinary Na as continuous variables and determined the associations of 100 mmol/d increase in urinary Na and change in BP using multilevel linear models. These modeling approaches were implemented separately for different urinary Na measurements.

All models included 3‐level random intercepts to account for multilevel clustering by the participant, participants within the household, and households within communities. We report findings of unadjusted models; models adjusted for age, sex, and BMI; and models additionally adjusted for smoking and alcohol consumption, physical activities, religion, hours of sleep, and household wealth score. Age and BMI were included as continuous covariates in models, whereas other covariates were included as categorical variables. Although we collected the information on consumption of table salt, we did not use it in the model to avoid possible collinearity with urinary Na. Religion was considered as a covariate because of differences in food across Hindus and Muslims—Hindus are often vegetarian and tend to eat less meat, but Muslims consume animal protein.^42^

Study participants diagnosed with hypertension or chronic kidney disease may receive advice from health professionals on a salt‐restricted diet. To avoid biased results because of this reverse causation, in sensitivity analyses, we restricted the analyses among participants who were not hypertensive, diabetic, and had no chronic kidney disease based on their self‐reported information.

To determine the multicollinearity problems in the regression models, we reported diagnostics for multicollinearity following the implementation of the fully adjusted linear models. For 3 types of urinary Na measurements, we reported variance inflation factors (VIFs) for the coefficients, tolerance, Leamer's statistics, and implemented Klein's rule. VIFs quantify how much the variances of the estimated coefficients are increased over the case of no correlations among the predictors, and tolerance is defined as 1/VIF.^43^, ^44^ Uncentered VIFs can more easily discover collinearity when constant terms are included.^45^ High VIF and low tolerance are suggestive of multicollinearity. Leamer's statistics is the square root of the ratio of variances of the estimated coefficient when estimated without and with other regressors^44^, ^46^—Leamer's statistics close to 1 means less correlation with regressors. The Klein's rule suggests the presence of multicollinearity if the $R_{j}^{2}$ of the auxiliary regression (eg, regression of the regressors on each other—$R_{j}^{2}$ will come from the regression of xj on other regressors) is greater than the overall *R* ^2^ (eg, regression of “Y” on all regressors).^44^ Statistical analyses were performed in Stata, version 15.0 and R, version 3.3.1.

### Ethical Approval

Informed written consent was taken from all the participating household members and the household heads. The study was approved by the Ethical Review Committee of International Centre for Diarrhoeal Disease Research, Bangladesh (icddr,b) (PR‐15096).

## Results

### Participants’ Characteristics

Mean age of the participants at enrollment was 42 (95% CI: 41–44) years, and mean BMI was 22 (95% CI: 21.5–22.3). Of these, 42% were male, 33% were smokers, and 99% had moderate work‐related physical activities (Table 1). All households used salt for cooking, but nearly 67% of participants reported consuming additional table salt with food. Of the participants, 15% were hypertensive, 7% had diabetes mellitus, and 4% had chronic kidney disease based on self‐reported information (Table 1).

**Table 1:** Characteristics of the Participants at Enrollment

Characteristics	All Participants (N=383)
Age, mean (95% CI)	42.2 (40.6, 43.7)
Male sex, % (n)	41.5 (159/383)
BMI, median (IQR)	21.5 (19.0, 24.6)
WHO BMI categories, % (n)	
Underweight (<18.5)	18.8 (72/383)
Normal weight (18.5 to <25)	60.3 (231/383)
Overweight (≥25 to <30)	17.5 (67/383)
Obese (≥30)	3.4 (13/383)
Smoking categories, % (n)	
Never	56.7 (217/383)
Former	10.2 (39/383)
Current	33.2 (127/383)
WHO work‐related physical activity, % (n)	
Sedentary	1 (4/383)
Moderate^a^	99 (379/383)
Vigorous^b^	0 (0/383)
Urinary creatinine (mg/d), median (IQR)	
Male	1420 (1050, 1744)
Female	1083 (902, 1262)
Household wealth categories, % (n)	
Lowest	23.5 (39/166)
Second	21.1 (35/166)
Third	20.5 (34/166)
Fourth	18.1 (30/166)
Highest	16.9 (28/166)
Added table salt with food	66.6 (255/383)
Added table salt during cooking, % (n)	100 (166/166)
Hours of sleep, % (n)	
<6 h	14.9 (57/383)
≥6 to <9 h	88.0 (280/383)
≥9 h	12.0 (46/383)
Alcohol consumption, % (n)	5.5 (21/383)
Religion, % (n)	
Hindu	29.2 (112/383)
Muslim	70.8 (271/383)
Self‐reported disease, % (n)	
Hypertension	15.1 (58/383)
Diabetes mellitus	6.5 (358/383)
Chronic kidney disease	3.9 (15/383)

BMI indicates body mass index; IQR, interquartile range; WHO, World Health Organization.

a Work involves moderate‐intensity activity that causes small increases in breathing or heart rate such as brisk walking (or carrying light loads) for at least 10 min continuously.

b Work involves vigorous‐intensity activity that causes large increases in breathing or heart rate (carrying or lifting heavy loads, digging or construction work) for at least 10 min continuously.

In the pre‐monsoon (enrollment) visit, we measured BP for 383 participants, and collected 24‐hour urine from 379, first morning urine from 308, and second morning urine from 383. During the monsoon visit, we measured BP for 359 participants, and collected 24‐hour urine from 354, first morning urine from 343, and second morning urine from 356. The mean systolic BP of the population was 111.13 (95% CI: 109.7, 112.6) mm Hg during pre‐monsoon and 110.6 (95% CI: 109.1, 112.1) mm Hg during monsoon (Table 2).

**Table 2:** Blood Pressure and Urinary Sodium (Na) Across Pre‐Monsoon and Monsoon Visit

Variables	Pre‐Monsoon	Monsoon	Both Visits
Systolic blood pressure, mean (95% CI)	111.13 (109.7, 112.6)	110.6 (109.1, 112.1)	110.9 (109.8, 111.9)
Diastolic blood pressure, mean (95% CI)	66.2 (65.5, 67.1)	66.6 (65.7, 67.6)	66.4 (65.8, 67.1)
Estimated daily Na from first morning urine sample, mean (SD)	121.8 (28)	122.1 (23)	121.8 (26)
Estimated daily Na from second morning urine sample, mean (SD)	121.2 (28)	123.2 (26)	122.2 (27)
Measured 24‐h urinary Na, mean (SD)	138.0 (69)	129.4 (71)	133.8 (70)

### Urinary Sodium

**Figure 2:**
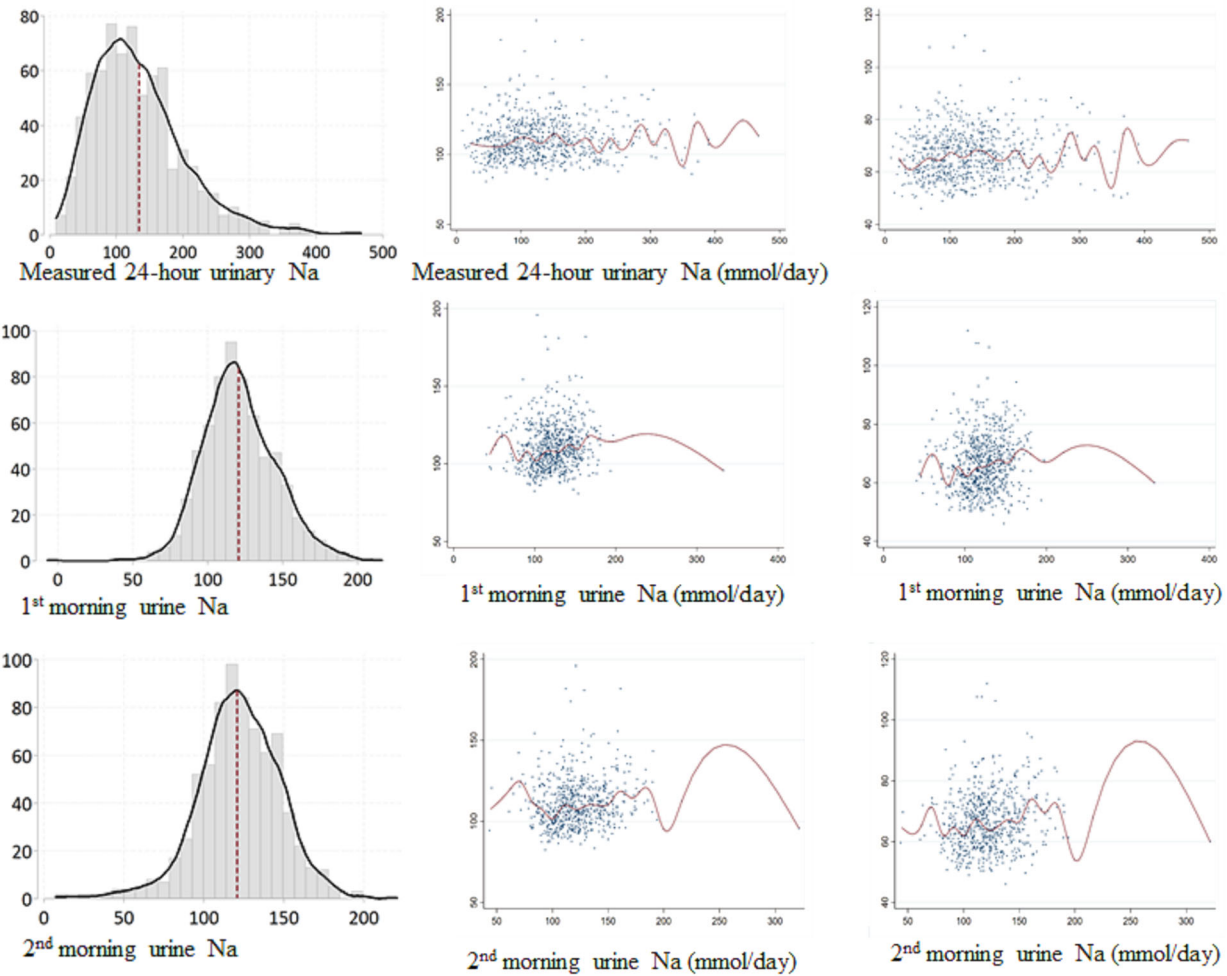
Histogram of 3 types of urinary Na measurements, and scatter plots with median spline graphs between the unadjusted relationship with blood pressure and 3 types of urinary Na measurements.

**Figure 3:**
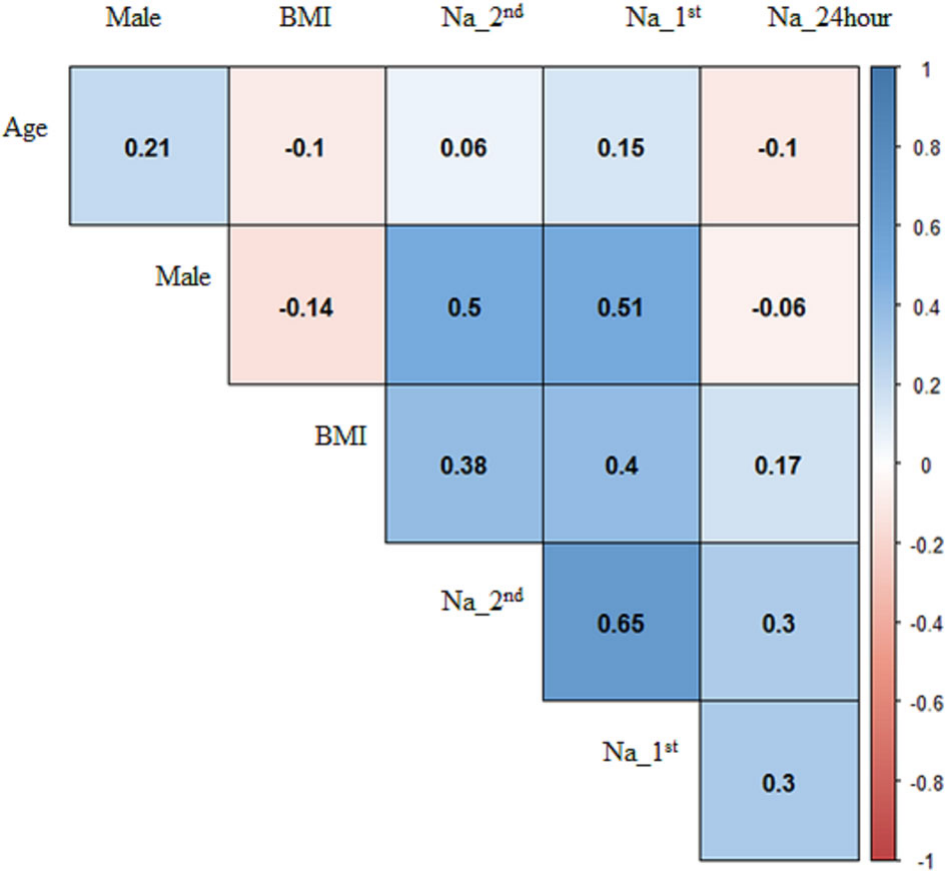
Correlation between 3 types of urinary Na measurements and age, sex, and BMI for the pooled samples from both visits. Na_^1st^: Daily estimated urinary Na from first morning urine samples. Na_^2nd^: Daily estimated urinary Na from first morning urine samples. Na_24hour: Daily measured urinary Na from first morning urine samples. BMI indicates body mass index.

In all person‐visits, the estimated daily Na was 122 (SD: 26) mmol/d from the first morning samples; 122 (SD: 27) mmol/d from the second morning samples; and 134 (SD: 70) mmol/d from the 24‐hour urinary samples (Table 2 and Figure 2). Men had higher estimated daily urinary Na from first (correlation coefficient [*r*_s_]=0.51) and second (*r*_s_=0.50) morning than the measured 24‐hour urinary Na (*r*_s_=−0.06) samples (Figure 3). Participants’ BMI had higher correlation with daily urinary Na estimated from first (*r*_s_=0.40) and second (*r*_s_=0.38) morning samples than the measured 24‐hour urinary Na (*r*_s_=0.17) (Figure 3). Participants’ intraclass correlation between urinary Na measures for the 2 visits were 0.52 for the estimated daily Na from first morning urine samples, 0.67 for the estimated daily Na from second morning urine samples, and 0.41 for the measured 24‐hour urinary Na.

### Urinary Na and BP Associations

**Figure 4:**
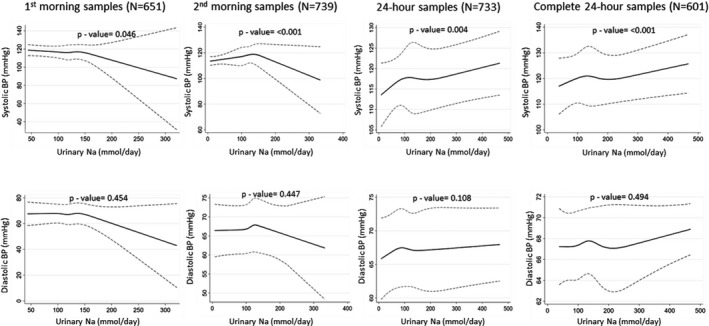
Restricted cubic spline plots (solid lines) and 95% CI (dashed lines) for urine sodium and blood pressure when adjusted for age, sex, body mass index, smoking status, physical activity, and household wealth. Restricted cubic splines were plotted with knots at fifth, 27.5th, 50th, 72.5th, and 95th percentiles. BP indicates blood pressure.

The restricted cubic spline plots illustrated no associations between urinary Na and BP up to 90th percentile (≈150 mmol/d) distribution of urinary Na, but then downward relationships with the increasing level of urinary Na for first morning urine samples (Figure 4). Spline plots from second morning urine samples resembled an inverted‐V shape. We found an overall upward and monotonic nonlinear relationship between 24‐hour urinary Na and BP (Figure 4). The similar upward monotonic relationship was also identified when the analyses were restricted among person‐visits of the complete 24‐hour samples. The plots for the relationship between urinary Na and DBP were almost similar to the SBP counterpart.

Compared with tertile 1 person‐visits of the first morning urinary Na, tertile 3 had −0.72 (95% CI: −2.41, 0.97) mm Hg difference in SBP in the fully adjusted models (Table 3). Compared with tertile 1 person‐visits of the second morning urinary Na, tertile 3 had 2.77 (95% CI: 0.41, 5.14) mm Hg difference in SBP in the fully adjusted models. Compared with tertile 1 person‐visits of the 24‐hour urinary Na, tertile 3 had 0.81 (95% CI: −1.92, 3.53) mm Hg difference in SBP in the fully adjusted models (Table 3). Each 100 mmol/d increase in urinary Na was associated with −3.83 (95% CI: −9.95, 2.29) mm Hg difference in mean SBP for the first morning samples; 1.53 (95% CI: −4.33, 7.40) mm Hg difference in mean SBP for the second morning samples; and 0.83 (95% CI: 0.00, 1.66) mm Hg difference in mean SBP for the 24‐hour samples (Table 4). Estimates for 24‐hour samples and complete 24‐hour samples were almost similar.

**Table 3:** Association Between Tertiles of Urinary Sodium (Na) and Blood Pressure in the Pooled Data From Both Visits

Measurement Methods	Systolic Blood Pressure			Diastolic Blood Pressure		
	Tertile 1	Tertile 2 β^a^ (95% CI)	Tertile 3 β^a^ (95% CI)	Tertile 1	Tertile 2 β^a^ (95% CI)	Tertile 3 β^a^ (95% CI)
Estimated daily Na+ from first morning urine sample (N=651)						
Unadjusted	Ref	0.33 (−1.33, 1.99)	0.81 (−1.69, 3.31)	Ref	0.28 (−0.38, 0.93)	0.28 (−1.48, 2.05)
Adjusted for visit, age, sex, and BMI	Ref	0.01 (−0.98, 0.99)	−0.64 (−2.29, 1.00)	Ref	0.02 (−1.01, 1.06)	−0.34 (−0.81, 0.13)
Full adjusted model^b^	Ref	0.04 (−1.00, 1.09)	−0.72 (−2.41, 0.97)	Ref	−0.04 (−1.00, 0.92)	−0.50 (−0.98, −0.02)
Estimated daily Na+ from second morning urine sample (N=739)						
Unadjusted	Ref	2.84 (1.45, 4.24)	3.22 (0.48, 5.96)	Ref	1.75 (0.60, 2.89)	1.58 (−0.61, 3.77)
Adjusted for visit, age, sex, and BMI	Ref	3.03 (1.32, 4.72)	2.81 (0.29, 5.33)	Ref	1.78 (0.43, 3.13)	1.44 (−1.78, 3.05)
Full adjusted model^b^	Ref	3.01 (1.31, 4.70)	2.77 (0.41, 5.14)	Ref	1.70 (0.42, 2.98)	1.34 (−0.19, 2.86)
Measured 24 h urinary Na+ (N=733)						
Unadjusted	Ref	0.71 (−2.12, 3.54)	0.56 (−2.10, 3.21)	Ref	−0.17 (−1.70, 1.37)	0.03 (−1.63, 1.70)
Adjusted for visit, age, sex, and BMI	Ref	0.83 (−2.26, 3.92)	0.85 (−2.26, 3.92)	Ref	−0.20 (−1.81, 1.41)	−0.13 (−1.71, 1.46)
Full adjusted model^b^	Ref	0.74 (−2.21, 3.70)	0.81 (−1.92, 3.53)	Ref	−0.21 (−1.79, 1.37)	−0.11 (−1.74, 1.51)
Complete measured 24 h urinary Na+ (N=733)						
Unadjusted	Ref	0.98 (−1.77, 3.72)	0.51 (−1.66, 3.72)	Ref	0.14 (−1.43, 1.71)	0.09 (−1.21, 1.39)
Adjusted for visit, age, sex, and BMI	Ref	1.55 (−1.55, 4.66)	1.25 (−1.26, 3.76)	Ref	0.22 (−1.38, 1.82)	0.04 (−1.20, 1.30)
Full adjusted model^b^	Ref	1.44 (−1.42, 4.31)	1.23 (−1.16, 3.62)	Ref	0.26 (−1.24, 1.77)	0.08 (−1.19, 1.35)

BMI indicates body mass index.

First morning void was collected on the second day and vice versa. Estimated daily Na+ from first morning urine; tertile 1: <110 mmol/d, tertile 2: ≥110 to <129 mmol/d; tertile 3: ≥129 to 322 mmol/d. Estimated daily Na+ from second morning urine; tertile 1: <112.5 mmol/d, tertile 2: ≥112.5 to <131.9 mmol/d; tertile 3: ≥131.9 to 333 mmol/d. Measured 24‐h urinary Na+; tertile 1: <96.2 mmol/d, tertile 2: ≥96.2 to <152.1 mmol/d; tertile 3: ≥152.1 to 468 mmol/d.

a Refers to differences in mean blood pressure (in mm Hg) of participants compared with reference category, obtained from linear regression.

b Model adjusted for visit, age, sex, BMI, physical activities and smoking status, alcohol consumption, sleep hours, religion, and household wealth.

**Table 4:** Association Between 3 Types of Urinary Sodium (Na) Measurements and Blood Pressure in the Pooled Data From Both Visits

Measurement Methods	Systolic Blood Pressure (β^a^ [95% CI])	Diastolic Blood Pressure (β^a^ [95% CI])
Estimated daily Na+ from first morning urine sample (N=651)		
Unadjusted	0.43 (−6.38, 7.24)	0.33 (−3.76, 4.42)
Adjusted for visit, age, sex, and BMI	−3.50 (−9.11, 2.10)	−2.23 (−5.23, 0.78)
Full adjusted model^b^	−3.83 (−9.95, 2.29)	−2.67 (−6.06, 0.72)
Estimated daily Na+ from second morning urine sample (N=739)		
Unadjusted	2.27 (−3.52, 8.07)	1.47 (−2.03, 4.97)
Adjusted for visit, age, sex, and BMI	1.58 (−4.22, 7.40)	0.74 (−2.19, 3.70)
Full adjusted model^b^	1.53 (−4.33, 7.40)	0.60 (−2.43, 3.64)
Measure 24 h urinary Na+ (N=733)		
Unadjusted	0.67 (−.22, 1.56)	0.25 (−.60. 1.09)
Adjusted for visit, age, sex, and BMI	0.87 (0.08, 1.66)	0.14 (−0.61, 0.88)
Full adjusted model^b^	0.83 (0.00, 1.66)	0.11 (−0.66, 0.88)
Complete measured 24 h urinary Na+ (N=601)		
Unadjusted	0.52 (0.07, 0.97)	0.15 (−0.29, 0.59)
Adjusted for visit, age, sex, and BMI	0.93 (0.57, 1.28)	0.08 (−0.33, 0.50)
Full adjusted model^b^	0.88 (0.56, 1.21)	0.06 (−0.33, 0.45)

First morning void was collected on the second day and vice versa. BMI indicates body mass index.

a Refers to differences in mean blood pressure (in mm Hg) of participants because of 100 mmol/d increase in urinary Na+.

b Model adjusted for visit, age, sex, BMI, physical activities and smoking status, alcohol consumption, sleep hours, religion, and household wealth.

**Figure 5:**
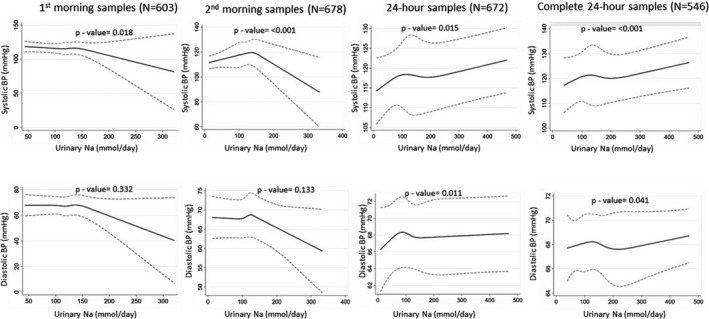
Sensitivity analyses excluding the self‐reported hypertension, diabetes mellitus, and chronic kidney disease participants. Restricted cubic spline plots (solid lines) and 95% CI (dashed lines) for urine sodium and blood pressure when adjusted for age, sex, body mass index, smoking status, physical activity and household wealth. Restricted cubic splines were plotted with knots at fifth, 27.5th, 50th, 72.5th, and 95th percentiles. BP indicates blood pressure.

Sensitivity analyses that excluded the self‐reported hypertensive, diabetic, and chronic kidney disease patients indicated little differences in estimates and their confidence intervals; however, the inferences and shape of relationship from the restricted cubic spline plots remained similar (Tables 5 and 6; Figure 5).

**Table 5:** Association Between Tertiles of Urinary Sodium (Na) and Blood Pressure After Excluding Participants With Self‐Reported Hypertension, Diabetes Mellitus, and Chronic Kidney Disease From the Pooled Data

Measurement Methods	Systolic Blood Pressure			Diastolic Blood Pressure		
	Tertile 1	Tertile 2 β^a^ (95% CI)	Tertile 3 β^a^ (95% CI)	Tertile 1	Tertile 2 β^a^ (95% CI)	Tertile 3 β^a^ (95% CI)
Estimated daily Na+ from first morning urine sample (N=603)						
Unadjusted	Ref	0.36 (−1.24, 1.97)	0.59 (−2.00, 3.18)	Ref	0.27 (−0.19, 0.74	0.28 (−1.62, 2.17)
Adjusted for visit, age, sex, and BMI	Ref	0.04 (−0.87, 0.95)	−0.76 (−2.70, 1.18)	Ref	0.04 (−0.91, 0.98)	−0.33 (−0.91, 0.98)
Full adjusted model^b^	Ref	0.13 (−0.99, 1.25)	−0.74 (−2.72, 1.23)	Ref	0.01 (−0.94, 0.96)	−0.43 (−1.22, 0.37)
Estimated daily Na+ from second morning urine sample (N=678)						
Unadjusted	Ref	2.92 (0.61, 5.2)	3.01 (−0.32, 6.33)	Ref	1.42 (−0.14, 2.98)	1.16 (−1.10, 3.42)
Adjusted for visit, age, sex, and BMI	Ref	3.07 (0.61, 5.52)	2.48 (−0.58, 5.54)	Ref	1.43 (−0.35, 3.20)	0.89 (−0.83, 2.62)
Full adjusted model^b^	Ref	3.07 (0.66, 5.47)	2.54 (−0.43, 5.51)	Ref	1.36 (−0.36, 3.08)	0.87 (−0.77, 2.51)
Measured 24 h urinary Na+ (N=672)						
Unadjusted	Ref	0.90 (−1.99, 3.79)	0.47 (−2.36, 3.32)	Ref	−0.16 (−1.72, 1.40)	−0.15 (−1.86, 1.57)
Adjusted for visit, age, sex, and BMI	Ref	1.04 (−2.18, 4.26)	0.73 (−2.23, 3.02)	Ref	−0.17 (−1.85, 1.52)	−0.30 (−2.02, 1.42)
Full adjusted model^b^	Ref	0.96 (−2.14, 4.07)	0.94 (−1.84, 3.72)	Ref	−0.17 (−1.85, 1.51)	−0.24 (−2.03, 1.56)
Complete measured 24 h urinary Na+ (N=546)						
Unadjusted	Ref	1.17 (−1.52, 3.86)	0.54 (−1.73, 2.81)	Ref	0.09 (−1.35, 1.53)	−0.10 (−1.24, 1.04)
Adjusted for visit, age, sex, and BMI	Ref	1.82 (−1.38, 5.03)	1.26 (−1.46, 3.98)	Ref	0.24 (−1.35, 1.84)	−0.10 (−1.31, 1.10)
Full adjusted model^b^	Ref	1.71 (−1.20, 4.63)	1.35 (−1.21, 3.91)	Ref	0.28 (−1.22, 1.79)	−0.03 (−1.23, 1.17)

First morning void was collected on the second day and vice versa. Estimated daily Na+ from first morning urine; tertile 1: <110 mmol/d, tertile 2: ≥110 to <129 mmol/d; tertile 3: ≥129 to 322 mmol/d. Estimated daily Na+ from second morning urine; tertile 1: <112.5 mmol/d, tertile 2: ≥112.5 to <131.9 mmol/d; tertile 3: ≥131.9 to 333 mmol/d. Measured 24 h urinary Na+; tertile 1: <96.2 mmol/d, tertile 2: ≥96.2 to <152.1 mmol/d; tertile 3: ≥152.1 to 468 mmol/d. BMI indicates body mass index.

a Refers to differences in mean blood pressure (in mm Hg) of participants compared with reference category, obtained from linear regression.

Model adjusted for visit, age, sex, BMI, physical activities and smoking status, alcohol consumption, sleep hours, religion, and household wealth.

**Table 6:** Association Between 3 Types of Urinary Sodium (Na) Measurements and Blood Pressure After Excluding Participants With Self‐Reported Hypertension, Diabetes Mellitus, and Chronic Kidney Disease From the Pooled Data

Measurement Methods	Systolic Blood Pressure β^a^ (95% CI)	Diastolic Blood Pressure β^a^ (95% CI)
Estimated daily Na+ from first morning urine sample (N=603)		
Unadjusted	−0.26 (−6.43, 5.91)	0.07 (−4.13, 4.27)
Adjusted for visit, age, sex, and BMI	−4.27 (−9.57, −1.03)	−2.60 (−5.99, 0.78)
Full adjusted model^b^	−4.39 (−10.22, 1.43)	−2.85 (−6.53, 0.83)
Estimated daily Na+ from second morning urine sample (N=678)		
Unadjusted	2.52 (−3.87, 8.92)	0.89 (−2.31, 4.10)
Adjusted for visit, age, sex, and BMI	1.63 (−4.80, 8.07)	−0.20 (−2.82, 2.42)
Full adjusted model^b^	1.68 (−4.98, 8.34)	−0.25 (−3.05, 2.55)
Measured 24 h urinary Na+ (N=672)		
Unadjusted	0.63 (−0.62, 1.88)	0.12 (−0.83, 1.06)
Adjusted for visit, age, sex, and BMI	0.81 (−0.41, 2.03)	0.01 (−0.88, 0.88)
Full adjusted model^b^	0.81 (−0.45, 2.08)	0.01 (−0.91, 0.92)
Complete measured 24 h urinary Na+ (N=546)		
Unadjusted	0.48 (−0.44, 1.41)	−0.03 (−0.56, 0.49)
Adjusted for visit, age, sex, and BMI	0.87 (−0.10, 1.84)	−0.10 (−0.67, 0.47)
Full adjusted model^b^	0.88 (−0.03, 1.79)	−0.10 (−0.61, 0.41)

First morning void was collected on the second day and vice versa. BMI indicates body mass index.

a Refers to differences in mean blood pressure (in mm Hg) of participants because of 100 mmol/d increase in urinary Na+.

b Model adjusted for visit, age, sex, BMI, physical activities and smoking status, alcohol consumption, sleep hours, religion, and household wealth.

The uncentered VIFs were 48.87 for the urinary Na from the first morning samples, 36.58 for the urinary Na from the second morning samples, and 4.93 for the measured 24‐hour Na (Table 7). Leamer's statistics close to 1 also suggests that 24‐hour urinary Na had the lowest multicollinearity. Klein's rule also suggests no collinearity for 24‐hour urinary Na, but presence of multicollinearity for the regression models of first and second morning urinary Na (Table 7).

**Table 7:** Multicollinearity Diagnostics for 3 Types of Urinary Sodium (Na) Measurements After Running the Fully Adjusted Linear Regression Model for Systolic Blood Pressure

Types of Urinary Na	Variance Inflation Factor (centered)	Variance Inflation Factor (uncentered)	Tolerance (centered)	Leamar's Method	Klein's Rule
Estimated daily Na+ from first morning urine sample	2.05	48.87	0.48	0.70	Multi‐collinearity present
Estimated daily Na+ from second morning urine sample	1.74	36.58	0.57	0.76	Multi‐collinearity present
Measured 24 h urinary Na+	1.06	4.93	0.94	0.97	No multi‐collinearity

## Discussion

We found that the association between Na intake and BP could diverge markedly depending on the methods used to estimate urine Na excretion. Based on different approaches of urinary Na measurement, we found different shapes of the relationship between Na intake and BP from the same population. The magnitude of the association also varied for different approaches of urinary Na measurement when the same statistical model was used. Therefore, it is important to consider the strengths of measurements of urinary Na before establishing the nature and magnitude of the association between Na intake and BP or other cardiovascular outcomes.

Multicollinearity occurs when variables in a multiple regression model are correlated (not perfect correlation),^47^, ^48^ which may provide biased coefficients of the estimates. We found higher correlations with the estimated daily urinary Na from first and second morning samples with sex and BMI compared with measured 24‐hour urinary Na. High correlations are intuitive for 2 reasons—(1) demographic variables are directly used in INTERSALT equations to estimate 24‐hour urinary Na; and (2) sex and BMI influence muscle mass, which is associated with urinary creatinine—a variable also used in INTERSALT equations. Creatinine is a waste product of muscle creatine, and ≈2% of muscle creatine is converted to creatinine every day.^49^ High BMI is an important risk factor for high BP, and males usually have high BP compared with females. Statistical models evaluating the Na intake (measured by urinary Na) and BP relationship are routinely adjusted for sex and BMI. Multicollinearity problems may arise if estimated urinary Na from spot urine samples are used and then adjusted for highly correlated BMI and sex in statistical models. Most multicollinearity diagnostics except Klein's rule suggested no multicollinearity in the regression model for the first and second morning urinary Na. However, all diagnostics suggested no or least multicollinearity for the regression model of measured 24‐hour urinary Na.

Twenty‐four‐hour urinary Na and BP relationship was upward and monotonic for the entire distribution. However, urinary Na and BP had a downward relationship beyond the 90th percentile distribution both for first and second morning urine samples. The confidence intervals for the estimates of the urinary Na from both first and second morning samples had wide ranges, which can be explained (1) because of multicollinearity, and (2) the 100 mmol/d increase for the urine Na from first and second morning samples is not biologically feasible because they had a very small SD. Therefore, estimation of daily Na from the first and second morning urine samples may not be a very good measure for capturing the variations of Na intake of the population. This is also evident as a higher intraclass correlation for the estimated daily Na from first and second morning urine samples, compared with the lower intraclass correlation for 24‐hour urinary Na. This may be particularly true since we used morning urine samples following overnight fasting. However, other studies have demonstrated that spot urine samples provide biased estimates irrespective of collection time, whether it is collected during morning, afternoon, or night.^21^, ^50^

Compared with the unadjusted models, regression estimates changed substantially after adjusting for age, sex, and BMI for the first and second morning Na samples, which suggests unstable model estimation.^48^, ^51^ In contrast, estimates from 24‐hour urinary Na had narrower confidence intervals, and relatively similar estimates following adjustments of age, sex, and BMI compared with the unadjusted models.

Our analyses have several important limitations. We are neither able to determine the causal association between Na intake and BP nor sense the mechanism by which Na intake may influence BP. Following a high Na‐containing diet, the plasma Na level increases, which in turn increases the blood volume and BP among salt‐sensitive individuals.^52^ There is growing evidence that a high Na‐containing diet increases the Na contents in the skin,^53^ which can influence capillary rarefaction and high peripheral resistance and high BP.^54^ Important regulatory mechanisms in response to the rise in blood volume and BP include rapid suppression of aldosterone excretion that facilitates renal excretion of Na,^15^ and pressure‐dependent natriuresis response of urinary Na excretion (usually nocturnal).^55^ Our 24‐hour urine sample collections from the participants at household‐level can be affected by over‐ and undercollection,^12^ which may bias estimates for 24‐hour urinary Na measures. Nevertheless, estimates from person‐visits of complete 24‐hour urine samples were almost similar. Our study was limited to a population from southwest coastal Bangladesh, but this result may differ in populations from other regions of the country and elsewhere. We used INTERSALT formulas, but it is recommended that every population have their own validated formula to estimate Na intake from the spot urine samples.^16^ Our linear models may not provide valid estimates in the presence of actual nonlinear relationships demonstrated by the restricted cubic spline models. However, we implemented linear models since the objective of our analyses was to compare the shape and magnitude of Na intake and BP relationship rather than identifying the valid estimates. We relied on sensitivity analyses based on participant‐reported information of diseases. Nevertheless, it is possible that participants may have had disease conditions but were unaware of this because they did not visit a physician.

We think 24‐hour urinary Na is the more appropriate measure of Na intake to determine the association with BP since this approach of Na intake measurement captures more variability of a population's daily sodium intake, and has less correlation with important demographic variables. Estimated Na from spot urine samples can only provide a mean estimation of Na intake, but will not capture the variability of population Na intake, and will likely provide biased estimation when used for determining the Na intake and BP associations.

## Sources of Funding

This research was funded by Wellcome Trust, UK, Our Planet, Our Health Award (Grant # 106871/Z/15/Z).

## Disclosures

None.
